# The side-entry method: An easy approach of umbilical vascular catheterization

**DOI:** 10.1016/j.resplu.2024.100694

**Published:** 2024-06-18

**Authors:** Mario Rüdiger, Jürgen Dinger

**Affiliations:** a Neonatology and Pediatric Critical Care Medicine, Department of Pediatrics, Faculty of Medicine and University Hospital Carl Gustav Carus, Technische Universität Dresden, Dresden, Germany; b Center for Feto / Neonatal Health, Faculty of Medicine and University Hospital Carl Gustav Carus, Technische Universität Dresden, Dresden, Germany

**Keywords:** Infant, Newborn, Umbilical artery, Umbilical vein, Catheter, Vascular access

## Abstract

Umbilical vascular catheterization remains an important technique in case a newly born infant requires resuscitation. Most textbooks recommend a complete transection of the umbilical cord and subsequent opening of vessel lumen with an iris forceps to place the catheter. That method, however, is challenging in emergencies.

Here we present an easy, quick and safe method of placing the umbilical catheters. The side-entry method could be an alternative to the conventional approach and is worth to enter pediatric textbooks and neonatal resuscitation guidelines.

Dear Editor,

A quick venous or arterial access might become necessary in newly born infants. Peripheral blood vessels are often difficult to access, central catheters are time-consuming procedures and an intra-osseous access is associated with severe side effects. Therefore, placement of catheters in umbilical vessels is the preferable approach and considered an essential skill of professionals treating neonates.

Most textbooks recommend a complete transection of the umbilical cord, thereafter vessel lumen is opened with an iris forceps to place the catheter.^1^ Since vessels are difficult to fix, the risk of bleeding increases, catheter placement can become difficult and often requires two practitioners. An alternative was described almost 40 years ago – the side-entry method,^2^ however that method is not part of recent resuscitation guidelines. The side-entry method is used in our institution for more than 30 years and we would like to raise the awareness of neonatal practitioner to that easy, quick and simple method that does require only one practitioner.

First steps of catheter placement are similar to the traditional approach: the abdomen and umbilical cord are prepared for sterile placement and an umbilical tie is placed around the cord just above the skin-level to prevent bleeding. The cord (clamped 2–4 cm from skin-level) is pulled on the clamp cranially or caudally for arterial or venous catheter placement, respectively (Fig. 1, *upper part*).

**Fig. 1:**
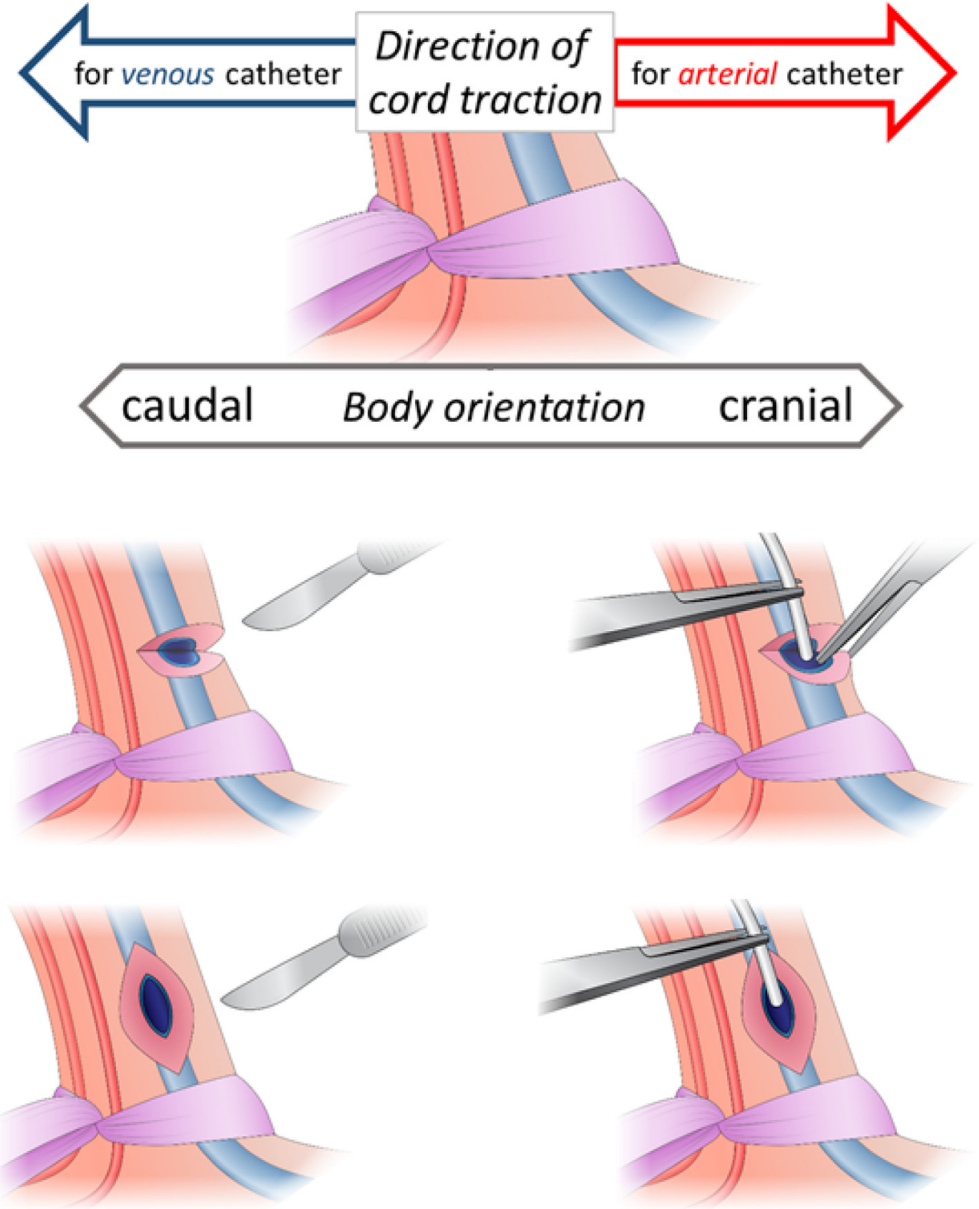
*Upper part:* The cord should be pulled cranially or caudally for arterial or venous catheter placement, respectively. *Middle part:* Horizontally performed incision. *Lower part:* Vertically performed incision.

Instead of a complete transection, the cord is bowed around the clamp to expose the vessel to cord surface. About 1–2 cm distally from the skin-level the cord is incised down to the vessels outer wall using a scalpel. The vessel wall is than carefully incised until the lumen is opened. Incision can be performed either horizontally or vertically (Fig. 1, *middle or lower part*). The catheter can be inserted and advanced to the appropriate position. Thereafter, a second catheter can be placed in another vessel if needed, using the same approach. Finally, the catheters can be fixed at the umbilical cord distally from the insertion place.

The side-entry method has been shown to be superior to the conventional method: more successful catheter placement, shorter time required for placement and less bleeding loss.^2^ In our experience, that method furthermore leaves the chance to enter the same vessel more proximally in the next attempt or to use the second artery for a later approach. Finally, it allows for an easy fixation at the remaining part of the umbilical cord.

An important advantage of the side-entry method is the need for only one practitioner. But another technique has been described as “fast, effective, and requires only 1 practitioner”.^3^ This method used the conventional approach of transecting the umbilical cord. Thereafter, a suture through the arterial wall is placed allowing for upward traction and complete control over the vessel-lumen. Even without any data the side-entry seems to be much easier.

In summary, the side-entry method – as an easy, quick and safe method of placing umbilical catheters – would be worth to be revived and to enter textbooks and neonatal resuscitation guidelines.

## Declaration of competing interest

The authors declare that they have no known competing financial interests or personal relationships that could have appeared to influence the work reported in this paper.
